# Gestosis Score in Antenatal Care: A Practical Approach to Pre-eclampsia Risk Assessment

**DOI:** 10.7759/cureus.93891

**Published:** 2025-10-05

**Authors:** Ishita Rathore, Meena T S, Preethika A, Minthami Sharon P

**Affiliations:** 1 Obstetrics and Gynaecology, Sree Balaji Medical College and Hospital, Chennai, IND

**Keywords:** fetal growth restriction, gestational hypertension, gestosis score, pre-eclampsia, risk assessment tools

## Abstract

Background

Gestosis score is a first-trimester screening tool developed by the Federation of Obstetric and Gynaecological Societies of India (FOGSI). This study aimed to evaluate the predictive accuracy of the gestosis score for identifying pregnant women at high risk of developing pre-eclampsia (PE) and associated adverse maternal and perinatal outcomes.

Methods

A prospective observational cohort study was conducted involving 130 pregnant women between nine and 12 weeks of gestation at a tertiary care hospital in Chennai, India. Participants were stratified into high-risk (GS ≥3) and low-risk (GS <3) groups based on clinical parameters, including BMI, mean arterial pressure, history of chronic hypertension, and use of assisted reproductive technology. Maternal and fetal outcomes were monitored until delivery.

Results

Of the 130 women, 39 (30%) had a GS ≥3, among whom 12 (30.7%) developed PE, compared to only 4 (4.4%) in the low-risk group (p<0.001). High GS was significantly associated with early-onset PE, intrauterine growth restriction, oligohydramnios, preterm delivery, lower gestational age at birth (36.4 vs. 38.2 weeks, p<0.001), reduced birth weight (2380 g vs. 2900 g, p<0.001), and higher rates of cesarean section and neonatal intensive care unit (NICU) admissions. Maternal age, obesity, anemia, chronic hypertension, and autoimmune disorders were strongly correlated with high GS. GS ≥3 demonstrated a sensitivity of 75% and a specificity of 76.3% for predicting PE.

Conclusion

GS is an effective, low-cost, and easily applicable tool for early identification of women at high risk for PE and related complications in resource-limited settings. Its integration into routine antenatal care may facilitate timely interventions and improve maternal and perinatal outcomes. Further multicenter studies are recommended to validate these findings and support broader adoption in national guidelines.

## Introduction

Hypertensive disorders of pregnancy (HDP) remain one of the leading causes of maternal and perinatal morbidity and mortality worldwide. Among these, pre-eclampsia (PE) is recognized as the most dangerous form, carrying substantial risks for both the mother and fetus. It is clinically characterized by new-onset hypertension after 20 weeks of gestation, typically accompanied by proteinuria or evidence of end-organ dysfunction. Globally, PE affects between 2% and 8% of all pregnancies and accounts for up to 19% of maternal deaths [1]. The burden is particularly pronounced in low- and middle-income countries, where limited access to early detection tools and timely management options exacerbates the severity of outcomes.

In India, the prevalence and consequences of PE remain alarming. Reports indicate an incidence of around 10.3%, with eclampsia contributing significantly to both maternal mortality and morbidity [2]. Despite policy-level progress, including increased institutional deliveries, challenges persist, particularly in rural and under-resourced areas where antenatal care services often fall short. The National Eclampsia Registry has further highlighted these concerns, not only documenting the high prevalence of HDP but also underscoring the issue of underreporting in peripheral healthcare facilities [3]. These data reveal an urgent need for strategies that allow for earlier identification and effective risk stratification of vulnerable women.

The pathogenesis of PE is complex and multifactorial, involving abnormal placentation, inadequate trophoblastic invasion, and systemic endothelial dysfunction, which collectively lead to widespread inflammatory responses and multiorgan involvement. Clinically, the disorder is associated with devastating maternal complications, including placental abruption, pulmonary edema, renal failure, and stroke. At the same time, adverse fetal outcomes such as intrauterine growth restriction (IUGR), preterm delivery, and stillbirth are frequently observed [4]. The timing of onset carries further prognostic importance: early-onset PE, defined as onset before 34 weeks of gestation, is associated with more severe maternal and neonatal complications compared to late-onset disease [5]. Thus, early detection is critical to improve both maternal and perinatal survival.

Current screening approaches for PE primarily rely on maternal demographic and clinical risk factors such as primigravidity, advanced maternal age, obesity, chronic hypertension, or a family history of hypertensive disorders in pregnancy. However, while these indicators provide some guidance, their predictive accuracy is often inadequate, especially when applied in isolation [6]. More advanced predictive tools, such as uterine artery Doppler studies and biochemical markers like placental growth factor (PlGF), have shown better accuracy in identifying high-risk women. Yet, the widespread application of these techniques remains limited in low-resource settings due to their high cost and the need for specialized equipment and expertise [7]. This disparity has created an unmet need for a reliable, affordable, and easily applicable risk assessment tool that can be implemented across various levels of the healthcare system.

Recognizing this gap, the Federation of Obstetric and Gynaecological Societies of India (FOGSI) introduced the gestosis score (GS) in 2019. Designed as a simple, first-trimester screening tool, the GS allocates weighted points (1, 2, or 3) to specific maternal clinical risk factors such as body mass index (BMI) above 30 kg/m², mean arterial pressure (MAP) greater than 85 mmHg, use of assisted reproductive technology (ART), and a prior history of chronic hypertension. Women who attain a total score of 3 or more are classified as being at high risk for developing PE. For these women, the guidelines recommend closer surveillance and the early initiation of prophylactic interventions, such as low-dose aspirin started before 16 weeks of gestation [8].

The major strength of the GS lies in its practicality and adaptability to the Indian healthcare landscape. Unlike biomarker-based tools, it does not require advanced laboratory facilities or specialized training, making it feasible to implement even at the primary healthcare level by nurses or health workers. Moreover, the score reflects risk factors that are particularly relevant to Indian women, including the growing prevalence of obesity, increasing use of ART, and the high background rates of comorbidities such as diabetes and anemia [9]. Thus, the tool offers a cost-effective, scalable solution that aligns with the realities of Indian maternal health services. 

Although the GS is already validated for predicting PE, its utility can vary across populations due to differences in demographics, comorbidities, and healthcare practices. This study was conducted in a South Indian tertiary care setting to assess its predictive value in a real-world, resource-limited context. The novelty lies in correlating GS-based risk stratification with actual maternal and fetal outcomes, providing locally relevant evidence to support its integration into routine antenatal care in similar healthcare environments.

Against this background, evaluating the effectiveness of the GS in predicting PE becomes critically important. By enabling the identification of high-risk women early in pregnancy, this tool has the potential to improve surveillance and allow for timely interventions that may reduce the burden of maternal and perinatal complications. Specifically, this study aimed to assess the ability of the GS to predict the subsequent development of PE in pregnant women and to analyze the maternal and fetal outcomes among high-risk women with scores ≥3.

## Materials and methods

This investigation was designed as a single-center, prospective observational cohort study conducted in the Department of Obstetrics and Gynaecology at Sree Balaji Medical College and Hospital (SBMCH), Chromepet, Chennai. The prospective cohort design was chosen because it allowed systematic enrollment of pregnant women at an early gestational age, risk stratification based on the GS, and longitudinal follow-up until delivery. Such an approach ensured that maternal risk factors and outcomes could be assessed in real time, thereby minimizing recall bias and enabling temporal association between the exposure, namely the GS, and the outcome, namely the development of PE.

Study setting and duration

The study was conducted at Sree Balaji Medical College and Hospital, a tertiary care teaching institution in Chennai that is equipped with advanced diagnostic and therapeutic facilities and caters to a wide patient base drawn from both urban and peri-urban regions, as well as referrals from peripheral and rural healthcare centers. This heterogeneity of the patient population improved the representativeness of the study. The duration of the study was from September 2023 to February 2025. Conducting the study over this extended timeframe allowed for the inclusion of multiple antenatal cycles and accounted for seasonal variations in maternal nutrition, infection patterns, and inter-individual physiological differences, thereby enhancing the robustness and generalizability of the findings.

Study population

The study population consisted of pregnant women presenting to the outpatient antenatal clinic or admitted via emergency services at the Department of Obstetrics and Gynaecology, SBMCH. Women between nine and 12 weeks of gestation were specifically targeted for enrollment, as this early window in pregnancy is considered critical for the application of risk assessment tools and for initiating preventive interventions such as low-dose aspirin therapy, which are known to be more effective when started before 16 weeks of gestation.

Inclusion and exclusion criteria

Women were eligible for participation if they had a singleton pregnancy and were between nine and 12 weeks of gestation at the time of presentation. Informed consent was obtained from all women willing to participate. No strict exclusion criteria were applied at enrollment to maximize the external validity and applicability of the study findings across diverse maternal profiles. However, women who were later diagnosed with major fetal anomalies during follow-up or who were unwilling to continue antenatal care at SBMCH were excluded from the final analysis to ensure consistency of data collection and reliable outcome tracking.

Sample size determination

Sample size was calculated for a prospective cohort design using the OpenEpi sample size calculator (Cohort/Unmatched, Version 3.01; Dean AG, Sullivan KM, Soe MM. OpenEpi: Open Source Epidemiologic Statistics for Public Health. www.OpenEpi.com). We specified a two-sided confidence level of 95% and 80% power to detect a clinically meaningful difference in PE incidence between exposed (GS ≥3) and unexposed (GS <3) groups. Based on existing literature [10] and local experience, we assumed an incidence of PE of ≈5% in the unexposed group and ≈20% in the exposed group. A ratio of unexposed to exposed close to 1:1 was used. With these inputs, OpenEpi returned a minimum required sample of 118 participants. Allowing for up to 10% loss to follow-up or incomplete data, the final target sample size was set at 130 participants.

Sampling method

A consecutive sampling method was employed, wherein every eligible pregnant woman attending the antenatal clinic during the study period and providing informed consent was enrolled sequentially until the target sample size of 130 participants was reached. Following recruitment, participants were categorized into two groups based on their GS assessed in the first trimester: those with a score ≥3 were assigned to the high-risk (exposed) group, while those with a score <3 were assigned to the low-risk (unexposed) group. Thus, while the initial recruitment followed a consecutive approach, group allocation was determined objectively by the screening tool, ensuring comparability between exposed and unexposed cohorts. This pragmatic approach minimized selection bias and mirrored real-world clinical practice in antenatal care settings.

Data collection protocol

Data collection commenced following informed consent, at which point a comprehensive baseline assessment was carried out. Sociodemographic details, including maternal age, education, occupation, and residential address, were recorded. Obstetric history was carefully elicited, including details of parity, previous pregnancies and their outcomes, mode of conception, and interpregnancy interval. A detailed medical history was also obtained, with attention to chronic illnesses such as hypertension, diabetes, thyroid disorders, and autoimmune conditions, all of which have relevance to the risk of PE.

Clinical examinations included anthropometric measurements, such as height and weight for the calculation of body mass index, and blood pressure assessment using a calibrated sphygmomanometer to calculate MAP. Laboratory investigations performed at the initial visit included hemoglobin estimation for anemia, thyroid function tests, fasting glucose levels for gestational diabetes, and autoimmune marker screening where clinically indicated. All assessments adhered to standardized hospital protocols to ensure consistency and reliability of data.

The GS was calculated at the first antenatal visit between nine and 12 weeks of gestation. The scoring system assigned weighted points to maternal parameters such as a body mass index above 30 kg/m², an MAP greater than 85 mmHg, a history of ART, and chronic hypertension. A score of 3 or more was designated as high risk for PE. Participants were therefore stratified into two groups: a high-risk cohort with a score equal to or greater than 3, and a low-risk cohort with a score below 3.

Follow-up strategy

All participants were followed until delivery, with structured antenatal surveillance. Women in the low-risk group were monitored through routine antenatal visits scheduled monthly until 28 weeks of gestation, fortnightly from 28 to 36 weeks, and weekly thereafter. At each visit, blood pressure, urine protein (through dipstick testing or quantitative analysis), weight gain, and clinical symptoms such as headache, visual disturbances, and epigastric pain were systematically assessed and documented.

Women in the high-risk cohort underwent more intensive monitoring. This included more frequent blood pressure checks, early ultrasonographic assessment of fetal growth, Doppler studies to evaluate uteroplacental circulation, and counselling regarding warning signs of PE. Preventive strategies such as lifestyle modifications, dietary advice, and weight management were emphasized. Prophylactic aspirin therapy was initiated where indicated, in line with current guidelines. To promote adherence to the follow-up schedule, participants received telephone reminders before each scheduled visit, and, where possible, transportation assistance was provided. Women who missed scheduled visits were contacted and encouraged to reschedule to maintain continuity of care and data collection.

Outcome measures

The primary outcome of interest was the incidence of PE among the study participants and its association with the baseline GS recorded in the first trimester. The predictive ability of the score was assessed by comparing the risk stratification at baseline with the actual development of PE during pregnancy. PE was diagnosed according to the criteria established by the American College of Obstetricians and Gynecologists, namely new-onset hypertension after 20 weeks of gestation accompanied by proteinuria or signs of end-organ dysfunction. Secondary outcomes encompassed both maternal and fetal measures. Maternal outcomes included the development of complications such as eclampsia, HELLP syndrome (hemolysis, elevated liver enzymes, and low platelets), fetal growth restriction, oligohydramnios, preterm delivery, mode of delivery, gestational age at delivery, and the need for intensive care admission. Fetal outcomes assessed included birth weight, Apgar score at 5 minutes, requirement for neonatal intensive care unit (NICU) admission, and neonatal mortality.

Statistical analysis

Data were initially entered into Microsoft Excel (Microsoft, Redmond, WA, USA) and then exported to MedCalc version 19.0 (MedCalc Software Ltd, Ostend, Belgium) for statistical processing. Descriptive statistics such as mean, median, standard deviation, and proportions were used to summarize baseline characteristics and outcomes. Categorical variables were compared using the chi-square test or Fisher’s exact test, while continuous variables were analyzed using the Student’s t-test or the Mann-Whitney U-test, depending on the distribution of data. Statistical significance was determined using a p-value of less than 0.05, and 95% confidence intervals were reported to enhance interpretability.

Ethical considerations

The study was conducted in strict accordance with the ethical principles outlined in the Declaration of Helsinki. The study protocol was reviewed and approved by the Institutional Ethics Committee of Sree Balaji Medical College and Hospital (002/SBMCH/IHEC/2023/2018), ensuring compliance with national and international ethical standards governing biomedical research. Informed consent was obtained from all participants after explaining the objectives, procedures, risks, and benefits of the study in their local language. Women were reassured that participation was entirely voluntary and that refusal or withdrawal would not affect the quality of medical care they received. Confidentiality of all data was maintained by anonymization, and access was restricted to the research team. All women identified with a GS ≥3 were promptly informed of their risk status. They received counseling regarding potential interventions, including low-dose aspirin prophylaxis, close antenatal monitoring, and lifestyle modifications. This approach ensured that high-risk women received appropriate guidance while allowing their voluntary participation in the study.

## Results

Among the 130 women included in the study, 24 (18.5%) were either <19 or >35 years of age, while the majority, 106 (81.5%), were between 19 and 35 years. Nearly two-fifths were primigravida [49 (37.7%)], and 81 (62.3%) were multigravida. Based on BMI distribution, 95 (73.1%) had BMI <30, 25 (19.2%) had BMI between 30 and 35, and 9 (6.9%) had BMI >35. Maternal anemia was observed in 60 (46.2%) women, whereas 70 (53.8%) were non-anemic. Elevated MAP (>85 mmHg) was seen in 45 (34.6%) women, while 85 (65.4%) had MAP ≤85 mmHg. Hypothyroidism was present in 17 (13.1%) women, while 113 (86.9%) were euthyroid. Gestational diabetes mellitus (GDM) was documented in 65 (50.0%) women. Multifetal pregnancies were rare, occurring in only 2 (1.5%) cases. A history of hypertensive disorders in previous pregnancies was noted in 12 (9.2%) women. Pregestational diabetes was reported in 8 (6.2%), and chronic hypertension in 19 (14.6%). Autoimmune disorders were observed in 4 (3.1%) women, while 126 (96.9%) had none (Table 1).

**Table 1:** Overall distribution of maternal and clinical parameters (N = 130) *WHO Classification of BMI. This table represents the overall distribution of maternal and clinical parameters among the study participants. BMI, body mass index; MAP, mean arterial pressure; HTN, hypertension.

Parameter	Category	n (%)
Maternal age	<19 or >35 years	24 (18.5%)
	19-35 years	106 (81.5%)
Gravida status	Primigravida	49 (37.7%)
	Multigravida	81 (62.3%)
BMI*	<30	95 (73.1%)
	30-35	25 (19.2%)
	>35	9 (6.9%)
Maternal anemia	Yes	60 (46.2%)
	No	70 (53.8%)
MAP	>85 mmHg	45 (34.6%)
	≤85 mmHg	85 (65.4%)
Hypothyroidism	Yes	17 (13.1%)
	No	113 (86.9%)
Gestational diabetes mellitus	Yes	65 (50.0%)
	No	65 (50.0%)
Multifetal pregnancy	Yes	2 (1.5%)
	No	128 (98.5%)
History of HTN in previous pregnancy	Yes	12 (9.2%)
	No	118 (90.8%)
Pregestational diabetes	Yes	8 (6.2%)
	No	122 (93.8%)
Chronic HTN	Yes	19 (14.6%)
	No	111 (85.4%)
Autoimmune disorder	Yes	4 (3.1%)
	No	126 (96.9%)

**Figure 1:**
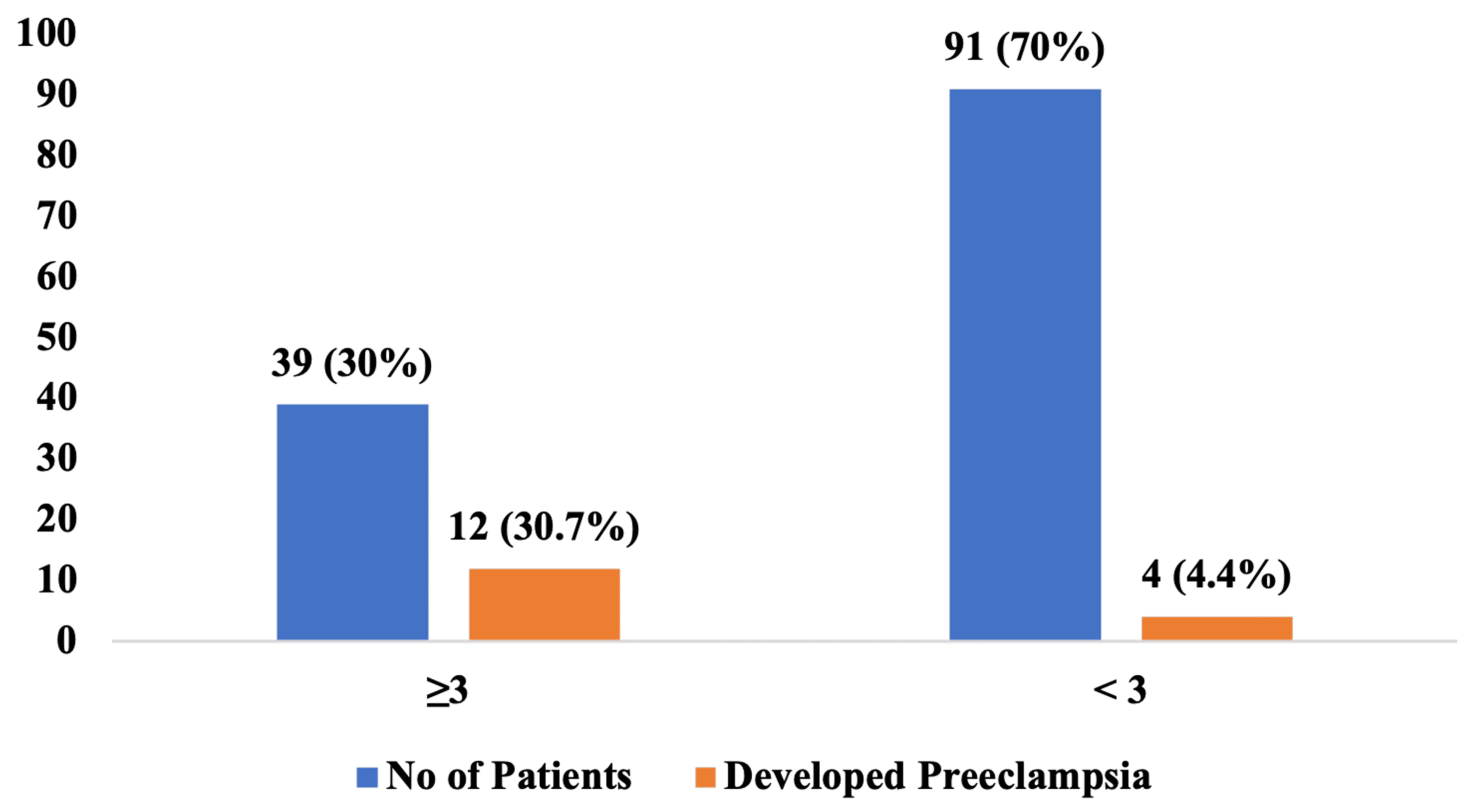
Distribution of gestosis scores among women with and without pre-eclampsia Data are represented as n (%). The data represent the total number of persons who developed pre-eclampsia among the participants grouped based on the gestosis score.

Out of 130 women assessed, 39 (30.0%) had a GS ≥3, among whom 12 (30.7%) developed PE. In contrast, of the 91 (70.0%) women with a GS <3, only 4 (4.4%) developed PE. Overall, 16 (12.3%) women in the study developed PE, and the association between higher GS (≥3) and development of PE was statistically significant (p < 0.001) (Figure 1). Out of the 130 women included in the study, 39 (30.0%) had a GS ≥3, of whom 12 (30.7%) developed PE, while among the 91 (70.0%) women with a score <3, only 4 (4.4%) developed the condition. The GS ≥3 demonstrated a sensitivity of 75% and a specificity of 76.3% for predicting PE. The positive predictive value (PPV) was 30.8%, indicating that nearly one-third of high-risk women developed PE, whereas the negative predictive value (NPV) was 95.6%, highlighting the tool’s strong ability to correctly identify women unlikely to develop the condition.

Among the 130 women studied, maternal age showed a significant association with high GSs, with 20 (83.3%) of those aged <19 or >35 years having GS ≥3 compared to 19 (17.9%) in the 19-35 years group (χ² = 34.5, p < 0.001). Primigravida status was more frequent in GS ≥3 (19, 38.8%) compared to multigravida (20, 24.7%), though not statistically significant (χ² = 3.1, p = 0.079). Higher BMI was strongly associated, as 9 (100%, n = 9) of women with BMI >35 and 10 (40.0%) with BMI 30-35 had GS ≥3, compared to only 19 (20.0%) with BMI <30 (χ² = 29.2, p < 0.001). Maternal anemia was also significant, with 31 (51.7%) anemic women having GS ≥3 versus 8 (11.4%) without anemia (χ² = 20.6, p < 0.001). Elevated MAP (>85 mmHg) was linked with GS ≥3 in 19 (42.2%) cases compared to 20 (23.5%) with lower MAP (χ² = 4.9, p = 0.026). Endocrine and metabolic conditions showed strong associations: hypothyroidism (11, 64.7% vs. 28, 24.8%; χ² = 10.6, p = 0.001), chronic hypertension (18, 94.7% vs. 21, 18.9%; χ² = 37.5, p < 0.001), pregestational diabetes (8, 100% vs. 31, 25.4%; χ² = 20.0, p < 0.001), and autoimmune disorders (4, 100% vs. 35, 27.8%; χ² = 7.9, p = 0.005). Multifetal pregnancies (2, 100%) and history of hypertensive disorders in previous pregnancies (12, 100%) were also significantly associated with GS ≥3 (χ² = 4.8, p = 0.028 and χ² = 24.7, p < 0.001, respectively). In contrast, GDM did not show a significant association, with 21 (32.3%)of GDM cases and 18 (27.7%) of non-GDM cases having GS ≥3 (χ² = 0.5, p = 0.497) (Table 2).

**Table 2:** Association of maternal and clinical variables with gestosis score among the study participants (n = 130) *Chi-square/Fisher's exact test. p-Value <0.05 is statistically significant. GS, gestosis score; BMI, body mass index; MAP, mean arterial pressure; HTN, hypertension.

Variables/Category	GS ≥3 (n = 39)	GS <3 (n = 91)	χ² value*	p-Value
Maternal age				
<19 or >35	20 (83.3%)	4 (16.7%)	34.5	<0.001
19-35	19 (17.9%)	87 (82.1%)		
Gravida				
Primigravida	19 (38.8%)	30 (61.2%)	3.1	0.079
Multigravida	20 (24.7%)	61 (75.3%)		
BMI				
<30	19 (20.0%)	76 (80.0%)	29.2	<0.001
30-35	10 (40.0%)	15 (60.0%)		
>35	9 (100.0%)	0 (0.0%)		
Maternal anemia				
Yes	31 (51.7%)	29 (48.3%)	20.6	<0.001
No	8 (11.4%)	62 (88.6%)		
MAP				
>85 mm Hg	19 (42.2%)	26 (57.8%)	4.9	0.026
≤85 mm Hg	20 (23.5%)	65 (76.5%)		
Hypothyroidism				
Yes	11 (64.7%)	6 (35.3%)	10.6	0.001
No	28 (24.8%)	85 (75.2%)		
Gestational diabetes mellitus				
Yes	21 (32.3%)	44 (67.7%)	0.5	0.497
No	18 (27.7%)	47 (72.3%)		
Multifetal pregnancy				
Yes	2 (100.0%)	0 (0.0%)	4.8	0.028
No	37 (28.9%)	91 (71.1%)		
History of HTN in previous pregnancy				
Yes	12 (100.0%)	0 (0.0%)	24.7	<0.001
No	27 (22.9%)	91 (77.1%)		
Pregestational diabetes				
Yes	8 (100.0%)	0 (0.0%)	20.0	<0.001
No	31 (25.4%)	91 (74.6%)		
Chronic HTN				
Yes	18 (94.7%)	1 (5.3%)	37.5	<0.001
No	21 (18.9%)	90 (81.1%)		
Autoimmune disorder				
Yes	4 (100.0%)	0 (0.0%)	7.9	0.005
No	35 (27.8%)	91 (72.2%)		

Among 130 women, PE developed in 12 (30.7%) with a GS ≥3 compared to 4 (4.4%) with a score <3 (χ² = 15.4, p < 0.001). Early-onset PE was more frequent in the higher-score group [5 (12.8%) vs. 1 (1.1%); χ² = 5.3, p = 0.021], although severity (severe vs. non-severe) did not differ significantly (p = 0.248). IUGR was observed in 6 (15.4%) of women with GS ≥3 versus 5 (5.5%) with GS <3 (χ² = 4.1, p = 0.042), and oligohydramnios in 5 (12.8%) vs. 3 (3.3%), respectively (χ² = 3.9, p = 0.048). Abnormal Doppler findings were noted in 4 (10.3%) of the high-score group compared to 2 (2.2%) of the low-score group, showing a trend toward significance (p = 0.056). Preterm delivery occurred more frequently among those with GS ≥3 [9 (23.1%) vs. 9 (9.9%), χ² = 3.1, p = 0.077]. Cesarean section was also more common in the higher-score group [21 (53.8%) vs. 32 (35.2%), χ² = 4.6, p = 0.032]. Importantly, the mean gestational age at delivery was lower in the high-score group (36.4 ± 1.5 weeks) compared to the low-score group (38.2 ± 1.8 weeks), which was statistically significant (t = 5.1, p < 0.001). The mean birth weight was significantly lower among neonates of women with GS ≥3 (2380 g ± 1.34) compared to those with GS <3 (2900 g ± 1.59, p < 0.001). An APGAR score <7 at 5 minutes was observed in 4 (10.3%) neonates in the GS ≥3 group versus 2 (2.2%) in the GS <3 group, though not statistically significant. NICU admissions were higher in the GS ≥3 group, with 5 (12.8%) cases compared to 3 (3.3%) in the GS <3 group (p = 0.023). No perinatal deaths were recorded in either group (Table 3).

**Table 3:** Association of gestosis score with maternal and perinatal outcomes among the study participants (N = 130) *Chi-square/Fisher's exact test for n (%). Independent-samples t-test for mean ± SD. p-Value <0.05 is statistically significant. GS, gestosis score; PE, pre-eclampsia; IUGR, intrauterine growth restriction; GA, gestational age; NICU, neonatal intensive care unit.

Variables	Category	GS ≥3 (n = 39)	GS <3 (n = 91)	χ²/t Value*	p-Value
Preeclampsia	Developed	12 (30.7%)	4 (4.4%)	17.2	<0.001
	Not developed	27 (69.3%)	87 (95.6%)		
Early-onset PE (n = 16)	Yes	5 (62.5%)	1 (12.5%)	5.3	0.021
	No	7 (37.5%)	3 (37.5%)		
Severity of PE	Severe	7 (87.5%)	1 (12.5%)	1.3	0.248
	Non-severe	5 (62.5%)	3 (37.5%)		
IUGR	Yes	6 (15.4%)	5 (5.5%)	4.1	0.042
	No	33 (84.6%)	86 (94.5%)		
Oligohydramnios	Yes	5 (12.8%)	3 (3.3%)	3.9	0.048
	No	34 (87.2%)	88 (96.7%)		
Abnormal Doppler	Yes	4 (10.3%)	2 (2.2%)	3.6	0.056
	No	35 (89.7%)	89 (97.8%)		
Preterm delivery	Yes	9 (23.1%)	9 (9.9%)	3.1	0.077
	No	30 (76.9%)	82 (90.1%)		
Mode of delivery	Cesarean	21 (53.8%)	32 (35.2%)	4.6	0.032
	Vaginal	18 (46.2%)	59 (64.8%)		
Mean GA at delivery (weeks)	–	36.4 ± 1.5	38.2 ± 1.8	t = 5.1	<0.001
Mean birth weight (g)	–	2380 ± 1.34	2900 ± 1.59	t = 16.4	<0.001
APGAR at 5 min	<7	4 (10.3%)	2 (2.2%)	2.4	0.121
	≥7	35 (89.7%)	89 (97.8%)		
NICU admissions	Yes	5 (12.8%)	3 (3.3%)	5.1	0.023
	No	34 (87.2%)	88 (96.7%)		
Perinatal death	Yes	0	0	–	–
	No	39 (100%)	91 (100%)		

## Discussion

The present study highlights the predictive accuracy of the GS as a cost-effective and clinically applicable screening tool for PE in antenatal women. Among 130 participants, 30.7% of those with a GS ≥3 developed PE compared to only 4.4% with GS <3, a statistically significant difference (p < 0.001). This finding demonstrates the ability of the score to stratify risk early in pregnancy and supports its use in routine obstetric care for early identification of high-risk women. Such risk stratification is particularly relevant in the Indian context, where PE incidence remains disproportionately high, estimated at 8-10% of pregnancies, largely due to maternal anemia, delayed antenatal registration, and undetected comorbidities [1]. International evidence increasingly supports multiparametric risk models. Cunningham et al. and James et al. highlighted that composite tools, incorporating maternal characteristics and hemodynamic indices, achieve higher predictive accuracy than single risk factors alone. Our study validates this approach, as multiple maternal and clinical variables were significantly associated with elevated GSs [2,3].

Maternal age emerged as a strong predictor. Women aged <19 or >35 years were more likely to have GS ≥3 (83.3%, p < 0.001). Extremes of age are known to impair vascular physiology, endothelial function, and maternal adaptation to pregnancy. Primigravidity was more frequent among GS ≥3 women (38.8%), although not statistically significant. This trend aligns with established evidence that nulliparity increases risk due to immunological maladaptation during first pregnancies. Obesity showed a robust association with higher scores. All women with BMI >35 had GS ≥3, and 40% of those with BMI between 30 and 35 were similarly classified as high risk (p < 0.001). Obesity contributes to low-grade inflammation, insulin resistance, and endothelial dysfunction, all of which impair placental development [2]. Recognizing this, the FOGSI guidelines have explicitly included obesity as a risk factor in the Gestosis scoring system [7]. Maternal anemia, another significant variable, was present in 51.7% of GS ≥3 women (p < 0.001). Anemia increases hemodynamic strain and can worsen placental hypoxia, a key mechanism in PE pathogenesis [1,6].

Hemodynamic indicators were also predictive. MAP >85 mmHg at booking was associated with GS ≥3 in 42.2% of women versus 23.5% in those with lower MAP (p = 0.026). This observation supports recommendations from ACOG and WHO that MAP in early pregnancy serves as a useful hemodynamic predictor of later PE [11,12]. Thyroid dysfunction was another relevant factor: 64.7% of hypothyroid women had GS ≥3 (p = 0.001). Hypothyroidism alters vascular reactivity and nitric oxide regulation, which predispose to hypertensive disorders [9]. Contrary to expectations, GDM was not significantly associated with high GS (p = 0.497). Since GDM often manifests later in pregnancy, its role in first-trimester-based risk prediction is limited [7]. In contrast, multifetal pregnancy was significantly associated (p = 0.028), consistent with the increased placental load and vascular demand. Strong associations were also observed with pregestational diabetes and chronic hypertension, where all affected women had GS ≥3 (p < 0.001). Both metabolic and vascular disorders predispose to placental dysfunction and oxidative stress, leading to early-onset disease [9,13]. Autoimmune disorders, such as systemic lupus erythematosus and antiphospholipid syndrome, were also significantly associated (100%, p = 0.005), consistent with their established role in defective placentation and impaired maternal-fetal immune tolerance [14,15].

Beyond maternal risk factors, the GS demonstrated predictive utility for maternal and fetal outcomes. Among women who developed PE, 75% belonged to the GS ≥3 group. Early-onset PE (<34 weeks) was more common in this group (62.5% vs. 12.5%, p = 0.021), underscoring the score’s time-sensitive predictive role. This aligns with Bartsch et al.’s systematic review, which confirmed that multiparameter models improve the prediction of early and severe PE [16]. The two-stage model of PE described by Roberts and Hubel [17] provides a mechanistic explanation, linking abnormal placentation in early pregnancy to systemic endothelial dysfunction in later stages, a cascade reflected in the Gestosis parameters.

Adverse fetal outcomes were also more frequent in GS ≥3 women. IUGR was observed in 15.4% compared to 5.5% in lower scores (p = 0.042), and oligohydramnios in 12.8% versus 3.3% (p = 0.048). These findings support the association of high scores with placental insufficiency-related complications, consistent with observations from Stamilio et al. and Abou-Nassar et al. [18,19]. Abnormal Doppler indices were also more frequent in GS ≥3 women (10.3% vs. 2.2%, p = 0.056), reflecting impaired uteroplacental perfusion [10]. Preterm delivery occurred in 23.1% of GS ≥3 women compared with 9.9% of those with lower scores, while mean gestational age at delivery was significantly lower (36.4 ± 1.5 weeks vs. 38.2 ± 1.8 weeks, p < 0.001). These findings highlight the role of GS in predicting not only PE but also its downstream obstetric complications. Delivery outcomes also reflected the stratification. Cesarean sections were more frequent among GS ≥3 women (53.8% vs. 35.2%, p = 0.032), reflecting higher rates of fetal compromise, abnormal Doppler findings, and maternal instability. Similar observations have been reported by Redman and Sargent [20] and Zeeman et al. [21]. Severe maternal complications, including HELLP syndrome and pulmonary edema, were also more frequently observed in high-score women, further validating the tool’s clinical relevance.

The overall findings of this study align with Indian and international literature. Upadhyay and Manhar et al. confirmed the high predictive value of GS ≥3 in Indian cohorts [4,5], while Gupta et al. and Khanijo et al. demonstrated that Gestosis achieves accuracy comparable to biomarker-based approaches [10,22]. Globally, this underscored the heavy burden of hypertensive disorders and the urgent need for low-cost, scalable screening tools, particularly in low-resource settings. The ISSHP and ACOG guidelines similarly emphasize integrating multiparametric clinical risk scores, such as Gestosis, into routine antenatal care to improve early detection [23,24].

This study has several limitations. It was restricted to a single-center cohort, which may limit generalizability. The relatively small sample size (n = 130) reduced power for subgroup analyses, particularly autoimmune and multifetal pregnancies. The absence of biochemical and Doppler biomarkers prevented direct comparison with multimodal screening approaches. Follow-up adherence depended on participant compliance, potentially introducing attrition bias. Finally, severe late pregnancy complications such as eclampsia were uncommon in this cohort, limiting insights into score performance for rare outcomes.

Despite these limitations, the study demonstrates that the GS is a strong predictor of PE and related complications in an Indian tertiary care setting. Its integration of region-specific maternal risk factors, affordability, and ease of use makes it well-suited for programmatic adoption in low-resource environments. Incorporating the GS into routine antenatal care could enable earlier identification of high-risk women, timely initiation of preventive measures such as low-dose aspirin, and closer surveillance for maternal and fetal complications. Large multicenter studies are recommended to further validate its performance and facilitate its inclusion into national guidelines for obstetric care.

## Conclusions

This study evaluated the utility of the FOGSI GS as a simple, cost-effective, and clinically relevant tool for first-trimester risk stratification. This study evaluated the utility of the GS as a simple, cost-effective, and clinically relevant tool for first-trimester risk stratification. A GS ≥3 was significantly associated with the development of PE, and importantly, with markers of disease severity, early onset, and adverse outcomes, including eclampsia, HELLP syndrome, pulmonary edema, IUGR, oligohydramnios, abnormal Doppler findings, and preterm birth. These associations confirm the prognostic value of the GS across the spectrum of hypertensive disorders in pregnancy. In conclusion, the GS emerges as a robust and practical screening tool that, if validated through larger multicenter studies, could be incorporated into national antenatal care guidelines to reduce the burden of hypertensive disorders and improve maternal-neonatal outcomes.
